# Roles of extracellular vesicles derived from healthy and obese adipose tissue in inter-organ crosstalk and potential clinical implication

**DOI:** 10.3389/fendo.2024.1409000

**Published:** 2024-08-29

**Authors:** Yue Han, Sheng Ye, Bowen Liu

**Affiliations:** ^1^ School of Engineering Medicine, Beihang University, Beijing, China; ^2^ Key Laboratory of Big Data-based Precision Medicine (Beihang University), Ministry of Industry and Information Technology, Beijing, China; ^3^ School of Medicine, The Chinese University of Hong Kong, Shenzhen, Guangdong, China; ^4^ School of Life Sciences, Westlake University, Hangzhou, China

**Keywords:** extracellular vesicles, adipose tissue, obesity, clinical implication, inter-organ communication

## Abstract

Extracellular vesicles (EVs) are nanovesicles containing bioactive molecules including proteins, nucleic acids and lipids that mediate intercellular and inter-organ communications, holding promise as potential therapeutics for multiple diseases. Adipose tissue (AT) serves as a dynamically distributed energy storage organ throughout the body, whose accumulation leads to obesity, a condition characterized by infiltration with abundant immune cells. Emerging evidence has illustrated that EVs secreted by AT are the novel class of adipokines that regulate the homeostasis between AT and peripheral organs. However, most of the studies focused on the investigations of EVs derived from adipocytes or adipose-derived stem cells (ADSCs), the summarization of functions in cellular and inter-organ crosstalk of EVs directly derived from adipose tissue (AT-EVs) are still limited. Here, we provide a systemic summary on the key components and functions of EVs derived from healthy adipose tissue, showing their significance on the tissue recovery and metabolic homeostasis regulation. Also, we discuss the harmful influences of EVs derived from obese adipose tissue on the distal organs. Furthermore, we elucidate the potential applications and constraints of EVs from healthy patients lipoaspirates as therapeutic agents, highlighting the potential of AT-EVs as a valuable biological material with broad prospects for future clinical use.

## 1 Introduction

EVs are small membrane-bound vesicles that released by almost all cell types. These vesicles carry the functional cargoes to their target cells, thus regulating intercellular and inter-organ crosstalk. EVs are crucial in the regulation of biological processes during physiological or pathological states, making them potentially clinically relevant. Especially, owing to their high biocompatibility, low immunogenicity, and extensive delivery range, EVs are being recognized as promising therapeutic agents for treating various diseases such as cardiovascular disorders and tumors.

Adipose tissue, a complex organ for energy storage, plays a central role in mediating systemic metabolism and maintaining energy homeostasis. The excessive accumulation of adipose tissue leads to obesity, which is a metabolic disorder characterized by chronic inflammation that triggers multiple diseases, including type 2 diabetes, heart disease and stroke (1). In addition to its primary function, adipose tissue has been well recognized to be an endocrine organ that produce adipokines, which regulate energy homeostasis and immune response (2, 3). In recent years, EVs from adipose tissue have been clarified as a novel category of adipokines. These EVs regulate systemic metabolism and distal organ function by delivering bioactive cargoes through autocrine, paracrine, and endocrine manners. Moreover, in obesity, the composition of EVs derived from adipose tissue are significantly altered, thus influencing the metabolic state and cell viability of recipient cells. To date, most studies on adipose tissue-derived EVs have focused on investigating the functions and applications of EVs derived from adipocytes or ADSCs. However, there is still a lack of systematic characterization and comprehensive summary regarding the composition and biological functions of EVs obtained directly from healthy or obese adipose tissue. Especially, given the accessibility of adipose tissue, evaluating the feasibility of utilizing AT-EVs as therapeutic agents could provide valuable insights for advancing their clinical applications.

In this review, we summarize the roles of EVs derived from healthy adipose tissue as mediators in intercellular and inter-organ crosstalk. We also discuss the roles of AT-EVs in facilitating the progression of multiple diseases under obesity. Moreover, we outline the procedures and principles of AT-EVs isolation for further investigation. Finally, we emphasize the composition and functions of lipoaspirate-EVs in biological processes, highlighting their potential for clinical translation.

## 2 Biogenesis and clinical application of EVs

**Figure 1:**
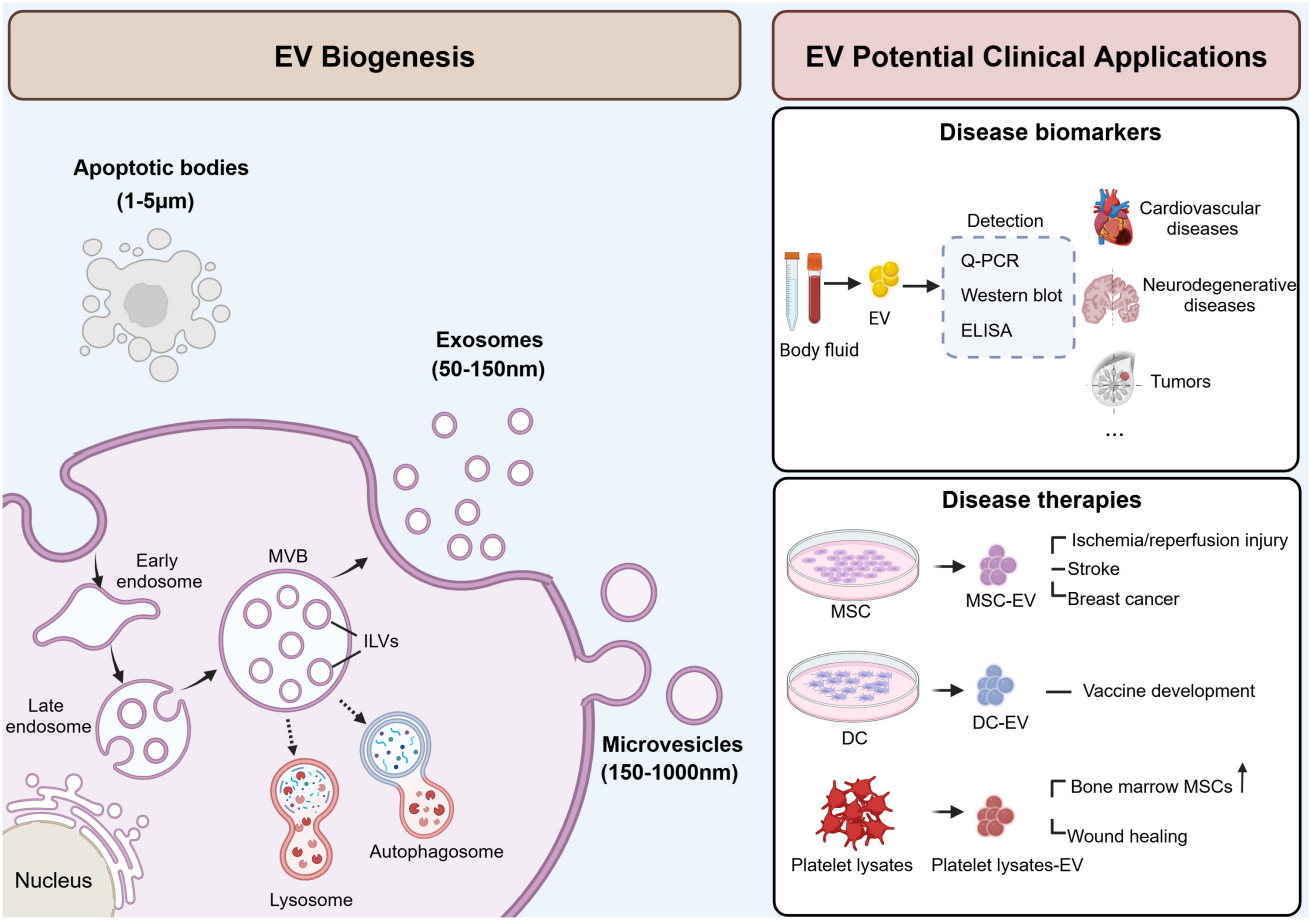
Schematic illustration of EV biogenesis and potential clinical applications. EVs are secreted into extracellular space and can be classified into three subgroups: exosomes (50-150 nm), microvesicles (150-1000 nm), and apoptotic bodies (1-5 µm). Exosomes are formed through the endocytic pathway and secreted by the fusion of MVBs with the plasma membrane. Microvesicles are formed directly from the plasma membrane. And apoptotic bodies are formed by cellular budding and the creation of apoptotic protrusions. EVs can be used as disease biomarkers and therapies. EVs derived from blood or body fluid carry specific cargoes that reflecting the state of parent cells, making them potential biomarkers for multiple diseases. Also, EVs derived from MSC, DC or platelet lysate also serve as promising therapeutic agents in various diseases. MVB, multivesicular body; ILV, intraluminal vesicles; ELISA, enzyme-linked immunosorbent assay; MSC, mesenchymal stem cells; DC, dendritic cells. This figure was created by using BioRender (https://biorender.com/).

Based on the biogenesis, EVs are primarily divided into three classes: exosomes, microvesicles, and apoptotic bodies (4) (**Figure 1**). Exosomes are small EVs (sEV) with diameter ranging from 50 to 150 nm, generated through the endocytic pathway, beginning as early endosomes formed by inward invagination of the plasma membrane. These early endosomes mature into late endosomes, which then form multivesicular bodies (MVBs). MVBs fuse with lysosomes or autophagosomes for degradation or with cell membrane to release the intraluminal vesicles (ILVs) as exosomes (5). Microvesicles, also referred to as large EVs (lEV) (6), ranging from 150 nm to over 1,000 nm, with an average size of 250-400 nm (7). They differ from exosomes as they are formed directly from the plasma membrane surface instead of originating from the endocytic pathway. Apoptotic bodies are the largest type of EVs, ranging in size from 1 to 5 µm. They form when programmed cell death is triggered by normal physiological signals or pathological processes, leading to cellular budding and the creation of apoptotic protrusions (5), and ultimately forming apoptotic bodies (5). Typically, apoptotic bodies are recognized by macrophages and cleared through phagocytosis (8).

In the physiological state, EVs serve as mediators that play a role in cell communication (9), tissue development and maintenance (10, 11), as well as immune response (12, 13). Meanwhile, EVs are also involved in the progression of diseases such as tumors, neurodegenerative diseases, and cardiovascular diseases. Due to their ability to transport disease-related biological information and their detectability in blood, EVs hold promise as potential diagnostic biomarkers. Crucially, active research is underway to harness the therapeutic potential of EVs for treating various diseases based on their biological functions. For example, EVs derived from mesenchymal stem cells (MSCs) have demonstrated therapeutic effects in models of myocardial ischemia/reperfusion injury (14), stroke (15), and breast cancer (16). Additionally, EVs from dendritic cells (DC) showed promise for vaccine development against infectious diseases such as tuberculosis (17). Moreover, EVs isolated from platelet lysates have been found to enhance the proliferation and migration of bone marrow-derived MSCs (18), and accelerate wound healing (19). EVs offer several advantages when used as therapeutic agents. Firstly, they exhibit low immunogenicity when employed in autologous applications, resulting in minimal potential side effects. Secondly, their lipid membrane composition facilitates fusion with target cells while preventing degradation. Finally, due to their modifiability, they can serve as efficient nanocarriers for loading functional miRNAs, thus enhancing the therapeutic efficacy. In conclusion, EVs hold promise in clinical applications, and the development of EV-related agents may provide therapeutic strategies for more diseases.

## 3 Healthy and obese adipose tissue

Adipose tissues are mainly categorized into two types, white adipose tissue (WAT) and brown adipose tissue (BAT). WAT is mainly composed of white adipocytes, with large unilocular/large lipid droplets and relatively few mitochondria (20). WAT maintains whole-body energy homeostasis by tightly regulating the balance of lipid storage and mobilization (21, 22). Additionally, WAT secretes a variety of adipokines, including adiponectin, leptin, omentin, resistin, as well as a variety of cytokines, such as tumor necrosis factor-α (TNF-α), IL-6, and monocyte chemoattractant protein-1 (MCP-1), all of which are essential for maintaining metabolic homeostasis in the body. WAT are classified into two types according to the location: subcutaneous adipose tissue (SAT) depot and visceral adipose tissue (VAT) depot (23). SAT is located in the abdomen, hip and thigh, and leg circumference. VAT is located surround the visceral organs, including pericardium, omentum, mesentery, retroperitoneum, and gonads (24). SAT is protective, whereas VAT located in intra-abdominal organs is detrimental due to its strong association with metabolic disorders, including obesity and diabetes (25).

Brown adipose tissue is more vascularized and sympathetic innervated, accounting for only a small portion (only ≈4.3%) of AT, and is mainly composed of brown adipocytes (26). Brown adipocytes contain many multilocular/small lipid droplets and significantly more cristae-dense mitochondria (27). Human BAT is located in the supraclavicular, axilla, neck, around the aorta, and in the subscapular region (28, 29). The primary function of BAT is mediating the non-shivering thermogenesis through uncoupling protein 1 (UCP1, also known as thermogenin), which is specifically expressed in brown adipocytes and located in the inner mitochondrial membrane (30). Recent studies have shown that beige adipose tissue, characterized by high mitochondrial content and the expression of UCP1, also participate in thermogenesis (31). In addition to regulating thermogenesis, BAT also secretes adipokines, known as brown adipokines or batokines, including metabolites, lipids, peptides, and miRNAs (32). These adipokines regulate energy balance and glucose homeostasis through crosstalk with various peripheral tissues, such as the liver, skeletal muscle, gut, central nervous system, and immune cells (33). Collectively, although the comprehensive understanding of BAT is still unclear, current evidence has demonstrated the great potential of BAT in the treatment of metabolic disorders *in vivo*.

Excessive accumulation of adipose tissue leads to obesity, which triggers numerous metabolic disorders such as type 2 diabetes, metabolic syndrome, and cardiovascular diseases (34, 35). In obese adipose tissue, mitochondrial function is significantly impaired, as evidenced by reduced oxidative metabolism (36, 37), compromised mitochondrial fatty acid oxidation (38), and decreased levels of glucose oxidation-related metabolites (39), leading to a decline in oxygen consumption and increased production of reactive oxygen species (ROS) (40). Adipokine levels are disrupted in obese adipose tissues, with decreased adiponectin and omentin, and increased cytokines including leptin, resistin, and FGF21 (41). These changes lead to chronic inflammation, characterized by elevated circulating IL-6 and TNFα (42, 43), indicating a pro-inflammatory phenotype in the immune microenvironment (44).

## 4 Adipose tissue-derived EVs

### 4.1 Cellular origin of adipose tissue-derived EVs

Although adipocytes are primarily responsible for fat storage, which is the main function of adipose tissue, they constitute only 15% to 30% of the total cell population. The majority of adipose tissue is composed of the stromal vascular fraction (SVF), which contains diverse cell types, including immune cells, fibroblasts, vascular cells, preadipocytes, and stem cells (45). The cellular origin of AT-EVs includes most of these cell types. Adipocyte-derived EVs are marked with adiponectin, resistin, perilipin A, and fatty acid binding protein 4 (FABP4) (46, 47). They induce the differentiation of monocytes into adipose tissue macrophages (ATMs) by secretion of mixed pro-inflammatory and anti-inflammatory cytokines (48). ADSC-EVs induce macrophages to adopt an anti-inflammatory phenotype, offering potential treatment for obesity-related metabolic dysregulation (49–51). EVs derived from ATMs also involve in regulating inflammatory responses and metabolic homeostasis. A study revealed that ATM-EVs carrying miRNAs was transferred to insulin target cells via paracrine or endocrine manners, significantly influencing insulin sensitivity and glucose homeostasis (52). Additionally, under the stimulation of glucagon, endothelial cells secrete EVs containing blood-derived signals that target adipocytes, participating in the response to systemic nutritional status changes (53).

In summary, AT-EVs are a heterogeneous mixture from various cell types, each carrying distinct cargoes and performing diverse functions. Many studies currently used adipocyte-derived EVs or ADSC-EVs to represent the components or functions of the AT-EVs. However, the crucial information relevant to the actual *in vivo* conditions are potentially overlooked. Therefore, to accurately understand EV functions and roles in physiology and pathology, it is essential to study EVs directly derived from adipose tissue.

### 4.2 Isolation of adipose tissue-derived EVs

**Figure 2:**
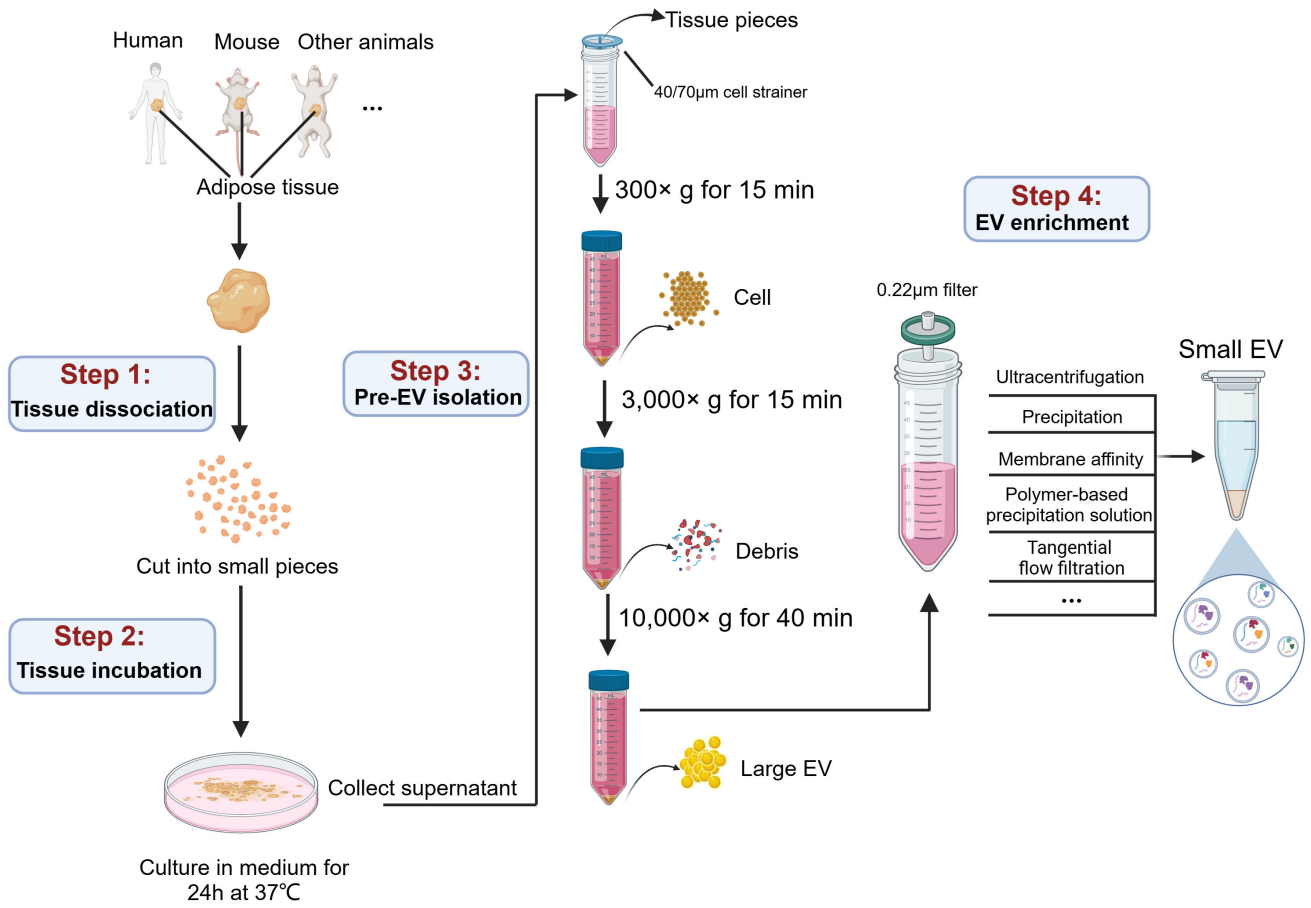
A graphic workflow for the isolation procedures of AT-EVs. The adipose tissues separated from human or other experimental animals were cut into small pieces on ice and incubated in medium for 24 h at 37 °C. Filtration and low force centrifugation (300 ×g for 15 min and 3,000 ×g for 15 min) were used to remove tissue pieces, cells and debris. After centrifugation at 10,000 ×g for 40 min, the large EVs were collected. The subsequent supernatant, after filtration through a 0.22 µm filter, the small EVs were isolated by multiple methods, including ultracentrifugation, precipitation, membrane affinity, polymer-based precipitation solution, and tangential flow filtration. This figure was created by using BioRender (https://biorender.com/).

Here, we collect the procedures of commonly used AT-EVs isolation in present studies (as shown in the **Table 1**), and further summarize the proper protocol for AT-EVs isolation (**Figure 2**).

**Table 1:** Summary of AT-EVs isolation procedures in previously published studies.

Species	Origin	Tissue dissociation	Tissue incubation	Pre-EV isolation	EV enrichment	Principle of EV enrichment	Citiation
Human or Mouse	——	Cut into small pieces	Culture in serum-free DMEM for 24 h	3,000 × g for 15 min; 10,000 × g for 30 min	110,000 × g for 70 min	Ultracentrifugation	(112)
Mouse	Interscapular region	Cut into extremely small pieces	Culture in serum-free DMEM for 24 h	3,000 × g for 15 min; 2,000 filter	ExoQuick-TC	Precipitation	(64)
Mouse	Visceral and subcutaneous adipose tissues	Cut into small pieces less than 0.1 cm^3^	Culture in serum-free DMEM for 24 h	1,000× g for 5 min; 10,000× g for 10 min; 0.22 μm filer	ExoQuick kit	Precipitation	(113)
Human	Omental and subcutaneous adipose tissue	Cut into small pieces	Culture in 199 medium (M199) supplemented with 1% FBS for 24 h.	2,000× g for 10 min	20,000× g for 70 min (for lEV)	——	(95)
Human	Paired omental and subcutaneous adipose tissue	Minced with scissors	Culture in 2% FBS/DMEM/F12 (1:1) + 1% penicillin/streptomycin for 18-24 h	70 μm filter; 500× g for 10 min; 10,000× g for 40 min	100,000× g for 90 min (×2)	Ultracentrifugation	(97)
Mouse	Interscapular (BAT) and inguinal area (WAT)	Minced into small pieces	Culture with α-MEM, 100 U/mL penicillin, and 100 μg/mL streptomycin in the Celstir Spinner Flask at 100 r/min for 2 days	300× g for 5 min; 2,000× g for 20 min; Ultracel-3 membrane at 5,000× g for 30 min; Ultracel-100 membrane at 5,000× g for 30 min	110,000× g for 70 min (×2)	Ultracentrifugation	(125)
Mouse	iWAT, eWAT, BAT	Sectioned into small pieces (1–2 mm)	Culture in α-MEM (without FBS) for 24 h	300× g for 10 min; 2,000× g for 20 min; 0.22 μm filter; 100,000× g for 120 min	exoEasy Maxi kit	Membrane affinity	(126)
Rats	Fat pads	Minced	Culture with Serum-free α-MEM in a Celstir spinner flask at 100 rpm for 48 h	2,000× g for 20 min; 40 μm filter; 0.22 μm filter; Ultracel-3 membrane at 5,000× g for 30 min; Ultracel-100 membrane at 5,000× g for 30 min	Total Exosome Isolation TM reagent	Polymer-based precipitation solution	(82)
Mouse	Interscapular brown adipose tissue	Cut into 2 mm^3^ pieces	Culture in DMEM containing 2% EV-free FBS, 0.5 mg/ml DNase I, 0.2 mg/ml RNase A and 1% P/S for 30 min; Medium was exchanged and culture for 24 h	300 × g for 10 min; 3,000 × g for 20 min; 10,000 × g for 30 min; 0.22 μm filter	110,000 × g for 70 min; 110,000 × g for 16 h	Ultracentrifugation	(84)
Rats	Inguinal adipose tissue	Cut into small pieces	Culture for 2 days	The supernatant was collected, filtered, and concentrated.	Total Exosome Isolation TM reagent	Polymer-based precipitation solution	(127)
Mouse	Interscapular region	Cut into small pieces	Culture in DMEM/F12 containing 10% exosome-free FBS for 48 h	The medium was collected.	ExoQuick-TC	Precipitation	(90)
Mouse	Visceral adipose tissue	Minced into small pieces (50–150 mm^3^)	Culture in serum-free conditioned medium for 48h	1,500 × g for 20 min	lEV: 13,000 × g for 1 h (×2); sEV: 100,000 × g for 1 h (×2)	Ultracentrifugation	(128)
Mouse	Visceral adipose tissue	Cut into small pieces less than 1 mm^3^	Culture in DMEM with 1% penicillin-streptomycin for less than 12 h	3,000× g for 15 min; 0.22 μm filter	100,000 × g for 2 h	Ultracentrifugation	(114)
Mouse	VAT: perirenal fat dissected from the fat pad surrounding the kidneys; SAT: bilateral superficial subcutaneous white adipose deposits between the skin and muscle fascia just anterior to the lower segment of the hind limbs. EAT: fat pad over the epididymis	Dissected	Culture in DMEM supplemented with 50 μg/ml gentamicin and 10% FBS with exosome-depleted fetal bovine serum	300× g for 10 min; 2,000× g for 10 min; 10,000× g for 30 min	100,000 × g for 70 min (×2)	Ultracentrifugation	(129)
Rats	PVAT from thoracic aorta	Cut into small pieces	Culture in DMEM supplemented with 10% exosome-free FBS for 24h	10,000 × g for 5 min; 0.22 µm filter	Total exosome isolation reagent	Polymer-based precipitation solution	(130)
Mouse	Visceral adipose tissue	Cut into small pieces less than 3mm	Culture in DMEM/F12 supplemented with Penicillin-Streptomycin and 10% fetal bovine serum with exosomes depleted for 12 h	500× g for 10 min, 2,000× g for 10 min; 10,000× g for 40 min; 0.22 μm filter	ExoQuick kit	Precipitation	(105)
Mouse	Visceral adipose tissue	——	Culture in DMEM for 6 h	——	exoEasy Maxi Kit	Membrane affinity	(98)
Mouse	Visceral adipose tissue	Cut into small pieces <4 mm	Culture in DMEM supplemented with 50 μg/ml gentamicin and 10% FBS with bovine sera exosomes predepleted	200 × g for 10 min; 500 × g for 10 min; 2,000 × g for 15 min; 10,000 × g for 30 min	70,000 × g for 60 min	Ultracentrifugation	(102)
Human	Adipose tissue	——	Culture in MEM supplemented with 100 U/mL penicillin, and 100 g/mL streptomycin and cultivated for 3 days at 100 r/min	——	Total exosome isolation reagent	Polymer-based precipitation solution	(81)
Human	Subcutaneous adipose tissue (from patients undergone a liposuction procedure)	Nonenzymatically processing by Lipogems device	——	800 × g for 30 min to remove large debris	KrosFlo Research 2i Tangential Flow Filtration System	Tangential flow filtration	(78)

Briefly, to obtain EVs from tissues as naturally as possible, a common approach involves isolating tissue explants from living tissues and culturing them *in vitro* for a short time (54). This approach results in the culture medium containing tissue-derived EVs, which is then isolated using techniques similar to those used for EVs from cell culture supernatants. Currently, this approach for isolating tissue-derived EVs has been applied to various tissues, including lung (55), brain (56, 57), heart (58), liver (59), muscle (60), tumor (61–63), as well as adipose tissue (64). However, the processing details vary depending on the specific characteristics of each tissue. Here we summarize the EV isolation procedure for adipose tissue, which involves four steps: tissue dissociation, tissue incubation, pre-EV isolation and EV enrichment.

#### 4.2.1 Tissue dissociation

To minimize the tissue degradation, fresh tissue samples should be immediately immersed in cold PBS after extraction from patients or animals, and then promptly transferred to the laboratory for subsequent experimental procedures (65). Since disrupting cellular integrity result in the release of intracellular vesicles that contaminates tissue-derived EVs, methods causing significant tissue damage, such as filtering (66, 67), vortexing (68), blending, or homogenization (66, 67), should be avoided. For adipose tissue, many studies performed tissue dissociation by directly cutting fresh adipose tissue into small slices (0.1 mm^3^) and minimizing the mechanical pressure applied to the tissue. This method is recommended for isolating EVs from various tissue types (69).

#### 4.2.2 Tissue incubation

Various enzymes, including collagenases (collagenase I, II, III, and IV) (70, 71), papain (72), and neutral protease (Dispase II) (55)are used to incubate with tissue for more thorough dispersion. In addition, DNase I is commonly added to prevent DNA aggregation (73). However, many studies on adipose tissue incubation use non-enzymatic treatments, which allows for the spontaneous release of EVs from the tissue interstitium. This gentler approach significantly minimizes damage to tissues and cells. Typically, this approach involves incubating sliced adipose tissue in a suitable culture medium for 24-48 hours under standard cell culture conditions, with a maximum incubation period of up to 72 hours.

#### 4.2.3 Pre-EV isolation

Following incubation, the majority of EVs in adipose tissue have been released into the culture medium, requiring subsequent enrichment of EVs from the supernatant. After removing the residual solid tissue fragments through 40 or 70 μm filters, the supernatants are subjected to differential centrifugation to remove cells and cell debris.

#### 4.2.4 EV enrichment

Large EVs can be precipitated at 10,000 ×g for 40 minutes. The supernatants were collected for the subsequent enrichment of small EVs. Previous studies have considered “ultracentrifugation” as the gold standard method for small EVs isolation (62, 74), and it is also widely recognized for isolating EVs from adipose tissue. However, due to inherent limitations such as incomplete removal of contaminating particles and time-consuming, alternative enrichment methods have been employed in many studies. These methods include precipitation (ExoQuick), membrane affinity (exoEasy Maxi kit) and polymer-based precipitation solution (Total Exosome Isolation TM reagent) (75). For high-throughput isolation, a clinically applicable, cell-free and sterile method of tangential flow filtration (TFF) has been established (76). TFF offers higher efficiency and can process large volumes (>1 L) of liquids, enabling large-scale production (77, 78). In addition, Casadei et al. have developed a novel cross-flow microfiltration method for isolating EVs from liposarcoma, a malignant tumor composed of adipocytes and other cell types (63, 79, 80). Subsequently, small EVs were pelleted and washed with PBS, followed by EV characterization assays, including size distribution determination by nanoparticle tracking analysis (NTA), morphology determination by transmission electron microscopy (TEM), and EV-specific molecules detection using immunoblotting or flow cytometry.

## 5 Roles of EVs derived from healthy and obese adipose tissue in systemic regulation

### 5.1 EVs derived from healthy adipose tissue

**Figure 3:**
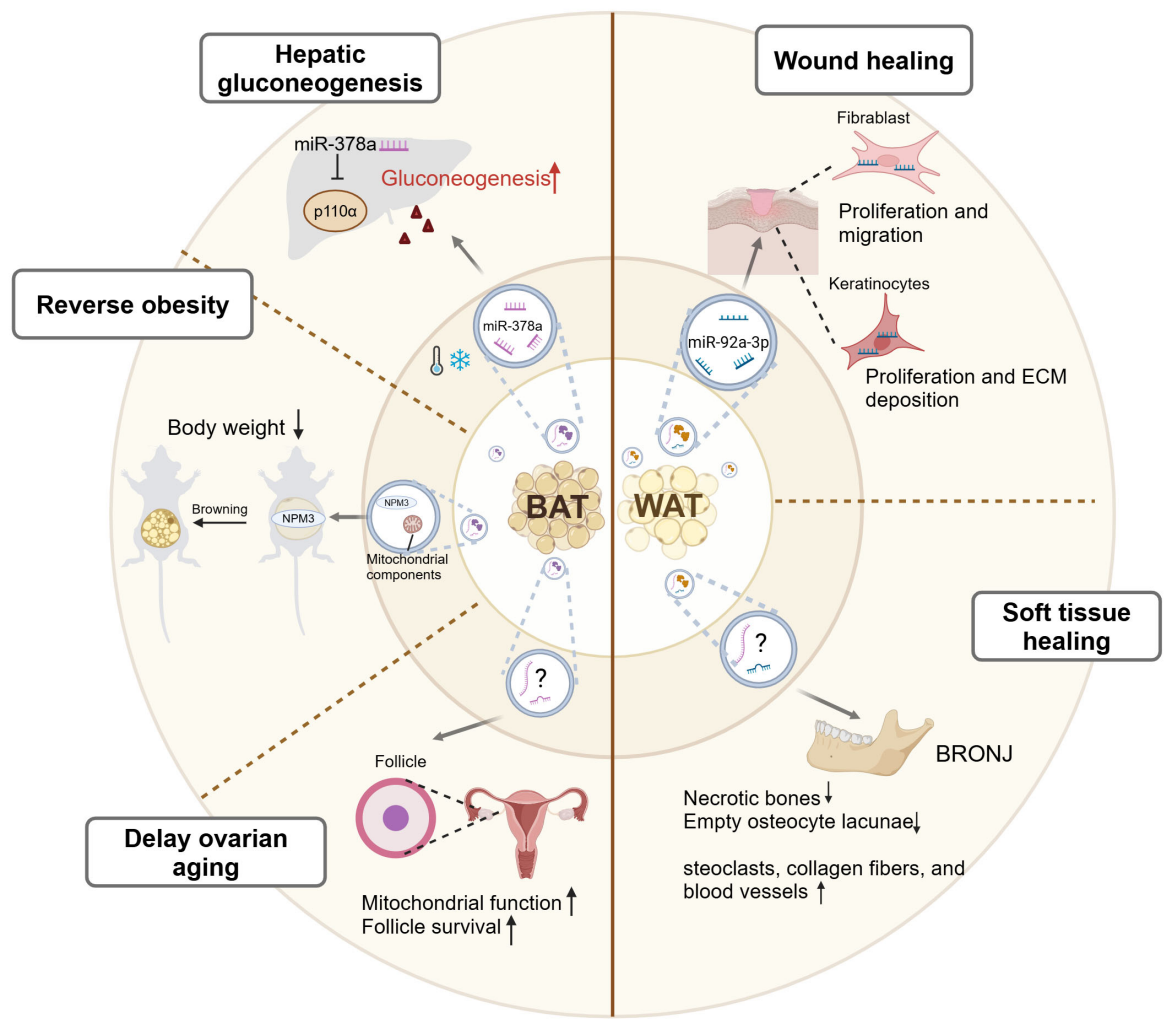
A graphic summary of the mediation of inter-organ crosstalk by BAT-EVs and WAT-EVs. Under cold stress conditions, BAT-EVs regulate systemic glucose metabolism by promoting hepatic gluconeogenesis through the effects of miR-378a. BAT-EVs also reverse obesity through two mechanisms. Firstly, their abundant mitochondrial components promote the oxygen consumption of recipient cells. Secondly, the NPM3 protein in BAT-EVs enhances the expression of browning related genes, thus inducing WAT browning. Additionally, BAT-EVs participate in the maintenance of reproductive system function by enhancing the mitochondrial function, thereby delaying ovarian aging. WAT-EVs improve skin wound healing through the effects of miR-92a-3p, which enhances the proliferation, migration, and ECM deposition of keratinocytes and fibroblasts. WAT-EVs also contribute to soft tissue healing in specific diseases, such as BRONJ. BAT, brown adipose tissue; WAT, white adipose tissue; NPM3, nucleophosmin3; ECM, extracellular matrix; BRONJ, bisphosphonate-related osteonecrosis of the jaw. This figure was created by using BioRender (https://biorender.com/).

Given their different origins, WAT-EVs and BAT-EVs exhibit different functions and roles, as shown in **Figure 3**. WAT is involved in tissue regeneration through secreting considerable amounts of adipokines, including cytokines and growth factors. Recently, evidence illustrated that WAT-EVs may also contribute to skin wound healing through releasing functional miRNAs. Shao et al. discovered that miR-92a-3p, abundant in WAT-EVs, targeted the large tumor suppressor kinase 2 (LATS2) in keratinocytes and fibroblasts (81). This activated the YAP/TAZ signaling pathway, enhancing the proliferation, migration, and extracellular matrix (ECM) deposition of keratinocytes and fibroblasts. And *in vivo*, WAT-EVs from adult patients undergoing skin flap transplantation treatment promoted wound healing in dorsal skin injured rats. Another example indicated that WAT-EVs promoted soft tissue healing. The WAT-EVs extracted from rat fat pad exhibited preventive effects against bisphosphonate-related osteonecrosis of the jaw (BRONJ) (82). In rats model, the WAT-EV-treated group showed reduced necrotic bones and empty osteocyte lacunae, along with an increase in osteoclasts, collagen fibers, and blood vessels. Moreover, WAT-EVs enhanced the proliferation, migration, and tube formation of human umbilical vein endothelial cells (HUVECs) inhibited by Zoledronic acid (Zol). These findings provide crucial theoretical foundations and insights for crafting innovative treatment strategies in wound healing processes.

BAT is the primary site of non-shivering thermogenesis (NST) in mammals and secretes numerous batokines that control energy metabolism and glucose homeostasis (83). Emerging evidence has shown that BAT-EVs also participate in the regulation of systemic homeostasis. Xu et al. demonstrated that cold-induced activation of BAT-EVs regulated systemic glucose metabolism by promoting hepatic gluconeogenesis (84). They identified that in mice model exposed to cold, miR-378a-3p was selectively packaged into BAT-EVs and delivered to the liver, as detected by *in vivo* visualization and tracking. Within hepatocytes, miR-378a-3p efficiently suppressed p110α, a key subunit of phosphoinositide 3-kinase (PI3K) that governed glucose metabolism (85), leading to increased glucose production. This study illustrated the intricate role of BAT-EVs in mediating inter-organ communication during cold exposure, thus contributing to the maintenance of systemic glucose metabolism stability (84). Apart from regulating whole-body glucose metabolism, BAT-EVs also participate in fat metabolism. Previous study has confirmed that BAT transplantation alleviated obesity and type I diabetes in various laboratories (86, 87). Zhou et al. discovered that BAT-EVs could also reverse obesity. BAT-EVs were enriched in mitochondrial components, which was consistent with BAT being mitochondria-rich (64). The uptake of BAT-EVs promoted the oxygen consumption of the recipient cells. Intravenous injection of BAT-EVs isolated from young healthy mice reduced body weight, lowered blood glucose levels, and restored cardiac function in high-fat-diet (HFD) mice (64). It is further evidenced by another study that BAT-EVs treatment induced weight reduction through activating the WAT browning. The cargo of BAT-EVs, nucleophosmin3 (NPM3), regulated the stability of PRDM16 mRNA, thereby enhancing the expressions of browning related genes, thus inducing WAT browning (88). This contributed to weight loss, increased energy expenditure, alleviated adipose tissue inflammation, and enhanced insulin sensitivity in HFD mice (88). Additionally, BAT-EVs also participate in the maintenance of reproductive system function. Ovarian aging is centrally drived by mitochondrial dysfunction (89). Zhang et al. demonstrated that injecting BAT-EVs that isolated from young female mice to aging mice enhanced mitochondrial function, promoted follicle survival, improved fertility, and extended ovarian lifespan, highlighting the significant role of BAT-EVs in delaying ovarian aging (90). In summary, BAT-EVs contribute to metabolic homeostasis and maintenance of distal organ functions through their cargoes, including miRNAs, proteins, and mitochondrial components.

Collectively, in healthy individuals, EVs released from both brown and white adipose tissue have a promising application prospect in metabolic regulation, organ function maintenance, and wound healing. Therefore, AT-EVs may be developed as a new cell-free therapy agent in the future. However, it should be noted that EVs derived directly from adipose tissue are a mixture of cellular EVs with complex compositions, which may contain components with opposing functions. Thus, their effects can be difficult to predict, and their roles in various diseases need further exploration.

### 5.2 EVs derived from obese adipose tissue

**Figure 4:**
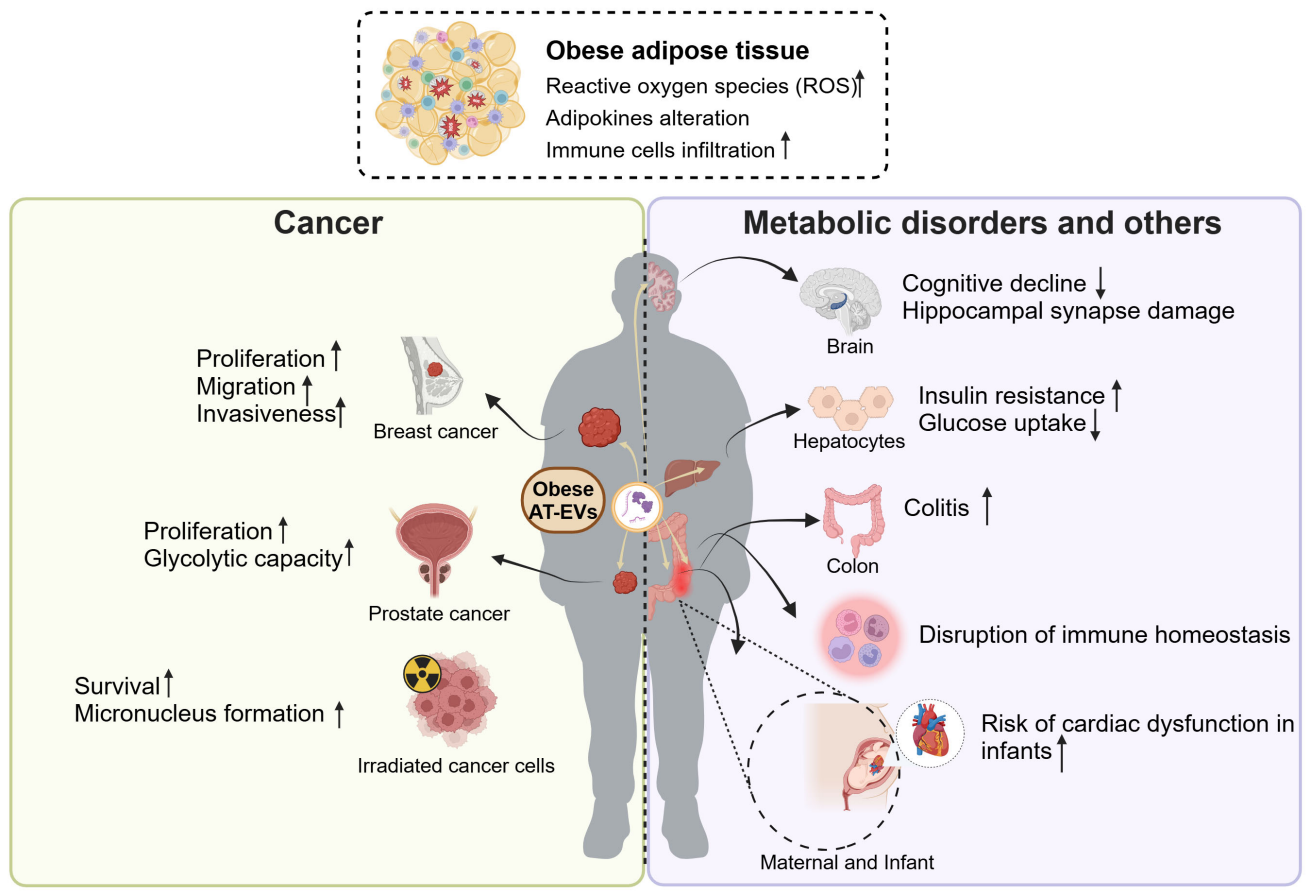
The roles of obese AT-EVs in cancer, metabolic disorders and other diseases. The obese adipose tissue exhibits elevated ROS, with adipokines alteration and abundant immune cells infiltration. The obese AT-EVs promote the progression of several types of cancers, including breast cancer and prostate cancer. Also, obese AT-EVs enhance the formation of micronucleus and promote the irradiated cancer cell survival. Additionally, obese AT-EVs can be transferred to distal organs to induce cognitive decline, insulin resistance, colitis, disruption of immune homeostasis and increasing risk of cardiac dysfunction in infants. ROS, reactive oxygen species; AT-EVs, adipose tissue-derived extracellular vesicles. This figure was created by using BioRender (https://biorender.com/).

Above evidence indicates that EVs derived from healthy adipose tissue serve as pivotal mediators for intercellular communication, facilitating cross-talk between adipose tissue and other organs. However, due to the heightened inflammation in obesity, such communication often yields unfavorable outcomes, including promoting the cancer progression, metabolic disorders, disruption of immune homeostasis, and distal organ dysfunction (**Figure 4**).

#### 5.2.1 Cancer progression

Obesity is increasingly recognized as a risk factor and a poor prognostic indicator for various types of cancer. It exacerbates distant metastasis, thereby amplifying disease severity and mortality rates (91–93). In addition to known mechanisms such as hormones, inflammation, and the immune system (94), emerging evidence suggests that obese AT-EVs are also involved in promoting cancer progression. A study revealed that obese AT-EVs promoted tumor activities and exacerbated the malignancy of breast cancer cells (95). Compared to AT-EVs isolated from lean patients, the AT-EVs from obese patients exhibited higher levels of leptin and MMP9 (closely associated with cell invasion) (96). The treatment of AT-EVs from obese patients enhanced the proliferation, migration, and invasiveness of breast cancer cells. Although this study had limitations, including the absence of *in vivo* experimental evidence and the unclear specific mechanism, these observations still offered evidence for the crucial role of obese AT-EVs in the tumor microenvironment. Another study demonstrated that obese AT-EVs induced the functional changes of tumors and exacerbated the malignant phenotype of prostate cancer. The EVs from paired omental (OM) and subcutaneous (SC) adipose tissue of patients enhanced the proliferation and glycolytic capacity of immortalized metastatic prostate cancer PC3ML cells by targeting the TWIST1/EMT signaling axis (97). Besides, it was currently found that obese AT-EVs promoted survival of irradiated m5S cells and enhanced micronucleus (MN) formation (98). This illustrated that in radiation therapy, obese AT-EVs promote the survival of irradiated cells with severe DNA damage, potentially leading to poor prognosis. This study partly explained the poor prognosis of obese patients in cancer treatment. Overall, obese AT-EVs contribute to tumor development and progression by increasing the risk of malignancies and leading to poorer prognoses.

#### 5.2.2 Metabolic disorders

Obesity often leads to insulin resistance, characterized by reduced insulin sensitivity in tissues such as the liver and muscles (99). In terms of adipose tissue accumulation site, VAT is more likely to induce insulin resistance compared to SAT (100, 101). Likewise, Deng et al. demonstrated that injecting VAT-EVs from obese mice intravenously induced insulin resistance in recipient mice (102). RBP4 carried by obese VAT-EVs activated macrophage in a paracrine manner and enhanced the production of proinflammatory cytokines IL-6 and TNF-α. The elevated TNF-α in obesity is a vital factor to induce insulin resistance because TNF-α activates IKK, p38 MAPK, JNK, and PKC, which directly target serine residues of insulin receptor substrate (IRS) (103, 104). Another study reported that obese VAT-EVs induced the insulin resistance in hepatocytes. The miR-141-3p in normal AT-EVs contributed to increasing AKT phosphorylation level in recipient cells, thereby promoting insulin signal transduction. While in obese VAT-EVs, the expression of miR-141-3p was decreased, resulting in impaired insulin signaling, and subsequently reducing insulin sensitivity and glucose uptake *in vitro (* 105). In conclusion, obese VAT-EVs serve as a detrimental mediator of intercellular communication, inducing insulin resistance either by recruiting immune cells to release cytokines or by directly secreting cargo molecules that target cells.

#### 5.2.3 Disruption of immune homeostasis

AT-EVs are a crucial source of circulating EVs, involved in immune regulation both inside or outside the adipose tissue microenvironment. Under the stress of obesity, the AT-EVs actively participate in regulating immune metabolic homeostasis or disruption by acting on nearby or distant immune cells. These obese AT-EVs were internalized into immune cells generally by endocytosis, membrane fusion, macropinocytosis, and phagocytosis (106–108). Obese AT-EVs carrying abundant TLR8 were internalized by monocytes and elevated the expression of CD16. This expression alteration subsequently boosted monocyte migration capacity, thereby facilitating leukocyte infiltration in the obese omental tissue (109). These findings may elucidate the roles of obese AT-EVs in immune cells infiltration and activation in obesity. In addition, emerging evidence has illustrated that obese AT-EVs contribute to modulating macrophage polarization. Obese AT-EVs contained higher level of miR-34a, which inhibited adipose-resident macrophages M2 polarization through repressing the expression of Krüppel-like factor 4 (Klf4), thus subsequently suppressing the anti-inflammatory phenotype (110). In addition, miR-155 in obese AT-EVs was upregulated, targeting SOCS1 and modulating the JAK/STAT signaling pathway to mediate M1 macrophage polarization (111). Taken together, the obese AT-EVs contained high heterogeneity of EV contents, which may have complex effects on the differentiation, migration and polarization of immune cells, thus disrupting the immune homeostasis.

#### 5.2.4 Distal organ dysfunction

In addition to affecting nearby organs through paracrine manner, obese AT-EVs can also influence the function of distal organs through endocrine manner. Obese AT-EVs can be remotely transported to the brain and promote insulin resistance related cognitive impairment. Wang et al. illustrated that injection of AT-EVs derived from HFD mice into recipient mice resulted in significant cognitive decline and hippocampal synapse reduction (112). The mechanism is that miR-9-3p in HFD-AT-EVs targeted BDNF (brain-derived neurotrophic factor, which is crucial for typical synaptic functions) in neurons, leading to synaptic damage. These observations may explain the declined brain functions in obesity. Wei et al. found that the VAT-EVs from HFD mice transferred to gastrointestinal system to aggravate the colitis severity in dextran sulfate sodium (DSS) model. The VAT-EVs loaded with miR-155 and other pro-inflammatory miRNAs induced M1 macrophage polarization, subsequently promoting DSS-induced colitis (113). This study showed that obesity changed the miRNAs in the AT-EVs, and transferred the EV phenotype from anti-inflammatory to pro-inflammatory. In addition to influencing different organs within independent individuals, VAT-EVs are also involved in the maternal-fetal communication, regulating placental development and fetal heart function (114). The miRNA profile in VAT-EVs from maternal obesity has been reported to be altered, notably with reduced levels of anti-inflammatory miR-19b. The injection of VAT-EVs from obesity promoted proinflammatory effects, leading to placental inflammation and fetal cardiac development defects (114).

In conclusion, adipose tissue has recently been recognized as an important endocrine organ in the body via active secretion of adipokines. AT-EVs act as a new type of adipokines, influencing the function and homeostasis of other organs. The miRNA or protein profile of AT-EVs were markedly altered in a high-fat diet and subsequent obesity, resulting in a transition from an anti-inflammatory to a pro-inflammatory phenotype in EVs. This phenotypic shift contributes to inflammatory infiltration and a certain level of function impairment. Additionally, targeting miRNAs altered in obese AT-EVs using specific inhibitors can effectively reverse the proinflammatory phenotype, offering a potentially promising therapeutic strategy in the future.

## 6 Potential clinical implication of adipose tissue-derived EVs

Currently, the most widely studied EVs for clinical applications in adipose tissue are ADSC-derived EVs, which have shown promising therapeutic effects. Recent research has revealed that ADSC-EVs primarily contribute to reducing inflammation, suppressing tumor proliferation and invasion (115, 116), inhibiting fibrosis progression (117), and promoting angiogenesis (118). At present, the isolation strategy for ADSC-EVs has been well-established, and the process can be summarized as follows: the extraction of adipose tissue from the body, separation of ADSCs, proliferation *in vitro* and purify EVs from the supernatant (119). However, this process is rather cumbersome and time-consuming. Currently, EVs isolated directly from adipose tissue represent a clinically feasible source of adipose tissue-derived EVs, offering a more cost-effective method with higher yields and greater efficiency compared to ADSC-EVs. Especially, Zhang et al. found that the therapeutic effects of AT-EVs surpassed those of ADSC-EVs due to the higher levels of functional miRNAs (120). As we mentioned above, AT-EVs from obese individuals may contain pro-inflammatory factors that promote disease progression. Therefore, only AT-EVs from healthy individuals are suitable as clinically effective therapeutic agents.

**Figure 5:**
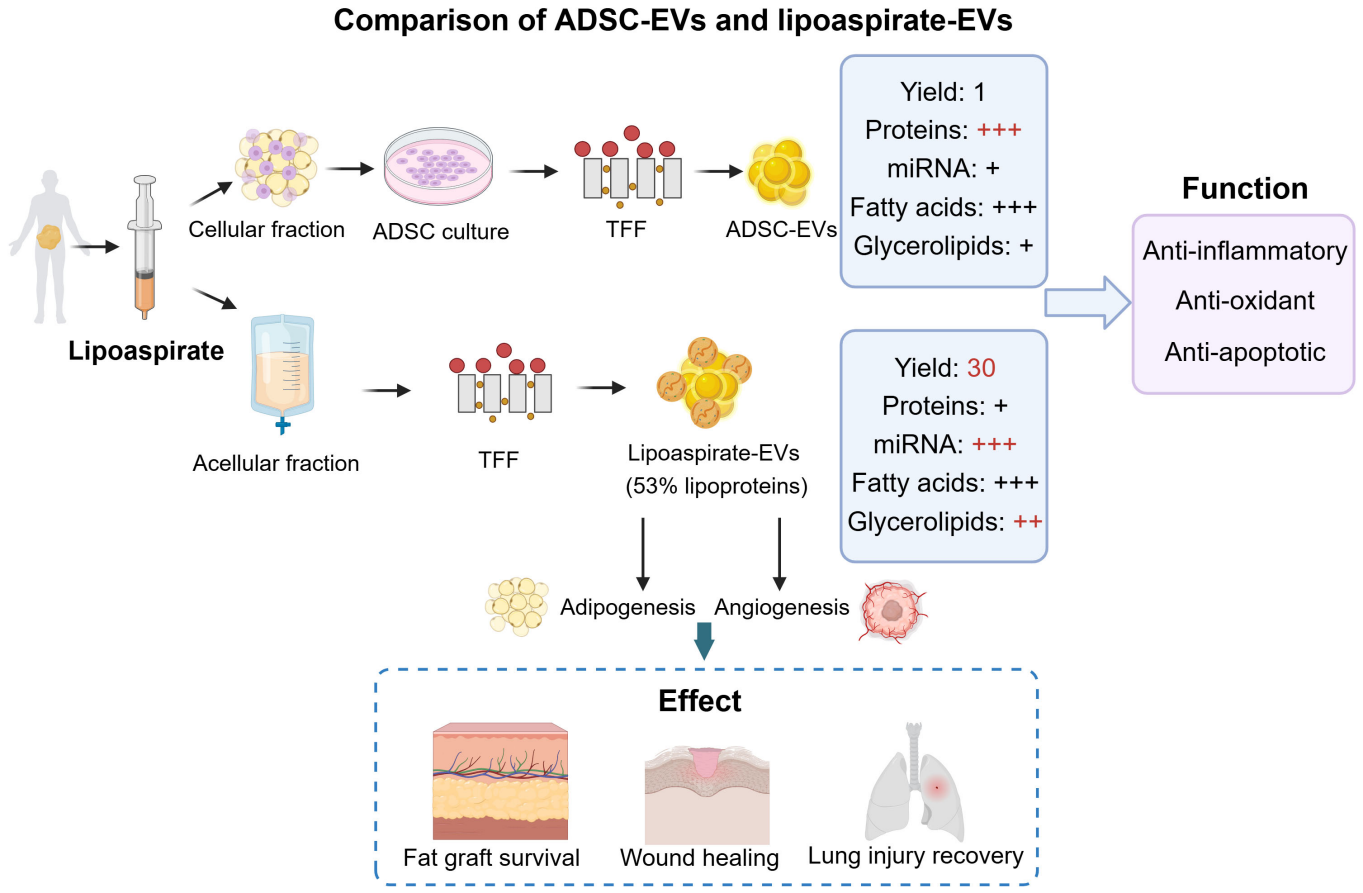
The comparison of ADSC-EVs and lipoaspirate-EVs. The ADSC-EVs were obtained from cell culture and lipoaspirate-EVs were obtained directly from acellular fraction. They vary in the yield, abundance of protein, miRNAs, fatty acids, and glycerolipids. ADSC-EVs and lipoaspirate-EVs both have the function of anti-inflammatory, anti-oxidant, and anti-apoptotic. Additionally, lipoaspirate-EVs have the therapeutic potential in promoting fat graft survival, wound healing, and lung injury recovery. ADSCs, adipose-derived stem cells; TFF, tangential flow filtration. This figure was created by using BioRender (https://biorender.com/).

Clinically, lipoaspirates obtained during liposuction represent the most accessible and abundant source of AT-EVs in the body. The isolation of lipoaspirate-EVs has been established. First, the lipoaspirate samples through liposuction from non-obese patients are mechanically dispersed by the non-enzymatic micro-fragmented process. After centrifugation to remove large debris, the EVs in the supernatant is isolated using method TFF. By using this isolation method, a study has compared the donor-matched ADSC-EVs and lipoaspirate-EVs in the EV characterization, yield, components, and function (78) (**Figure 5**). Both ADSC-EVs and lipoaspirate-EVs had the normal EV structure and size distribution. Notably, lipoaspirate-EVs samples contained lipoprotein-like spherical structures, which was confirmed by size-exclusion chromatography (SCE) that approximately 53% of the nanoparticles was lipoproteins (78). The EV yield from lipoaspirate was approximately 30-fold higher than that of cultured ADSCs (78). In terms of EV composition, the protein level was much higher in ADSC-EVs than lipoaspirate-EVs, however, the latter contained more miRNAs and glycerolipids (triglycerides in particular) (78). The ADSC-EVs and lipoaspirate-EVs exhibited similar protective and anti-inflammatory effects in macrophages. In conclusion, lipoaspirate-EVs are high-yielding, readily available, and possess fundamental EV characteristics, making them promising for large-scale clinical production and application.

Growing evidence suggests that lipoaspirate-EVs have significant effects on tissue recovery. For example, it was reported that lipoaspirate-EVs effectively promoted the proliferation and migratory ability of endothelial cells, thus promoting graft survival in a fat grafted mouse model (121). In the same year, another team found that lipoaspirate-EVs promoted angiogenesis of vascular endothelial cells and adipogenic differentiation of ADSCs *in vitro* and *in vivo*, resulting in improving the fat retention (122). These studies suggest that lipoaspirate-EVs have great potential as a new autologous component to aid in fat grafting. Furthermore, Wu et al. have developed a novel method of cell-free and EV-riched products (lipoaspirate fluid derived factors and extracellular vesicles, LF-FVs) extracted from human lipoaspirate (123). *In vitro*, LF-FVs promoted the proliferation and migration of fibroblasts. *In vivo*, LF-FVs significantly accelerated burn wound healing in rats, improved skin appendage regeneration, and inhibited scar formation. This study indicates that LF-FVs have potential applications in clinical wound healing. Additionally, Yu et al. found that lipoaspirate-EVs was a new type of bioactive substance to protect the microvascular barrier in mice (124). By intravenous injection, lipoaspirate-EVs protected the pulmonary microvascular endothelial cells (PMVEC) barrier integrity, thus reducing lung injury. Taken together, lipoaspirate-EVs, as a novel biomaterial, can be considered as a viable alternative to ADSC-EVs for clinical applications.

## 7 Conclusions and future perspectives

Emerging evidence from both *in vitro* and *in vivo* research has identified AT-EVs as crucial mediators of intercellular communication within adipose tissue through paracrine signaling, and they also participate in inter-organ crosstalk via endocrine signaling. These processes contribute to the maintenance of metabolism, immune response, and tissue repair. However, excessive accumulation of adipose tissue induces chronic inflammation, and the EVs released from this environment lead to adverse effects, including tumor progression, insulin resistance, and dysfunction of other organs. Numerous studies have demonstrated the promising clinical translational potential of AT-EVs. Lipoaspirates extracted from clinic serve as an excellent source of AT-EVs due to its high-yield and efficient isolation. It facilitates both adipogenesis and angiogenesis, making it applicable in the treatment of numerous diseases. To advance the clinical applications of AT-EVs, many challenges remain to be thoroughly explored. First, the sorting mechanism of AT-EV cargoes, particularly under physiological and pathological conditions, remains unclear. A comprehensive understanding of the sorting mechanisms aids in directing the loading of functional molecules into EVs. Second, in the clinical application of lipoaspirate-EVs, it is crucial to evaluate the impact of additional contaminants, such as lipoproteins, on treatment outcomes. Developing a more refined EV isolation method that maximizes purity without affecting yield holds promise for enhancing therapeutic efficacy. Furthermore, investigating appropriate storage methods that preserve the activity of AT-EVs, particularly their mitochondrial components, enhances the potential for future large-scale production applications. Such research has already been initiated in the field. Moreover, exploring the potential of lipoaspirate-EVs as carriers for functional miRNA delivery and to develop strategies for enhancing EV specificity is important. This makes lipoaspirate-EVs a valuable tool in precision medicine and targeted therapy. In conclusion, further advancements in AT-EVs research will provide a more comprehensive understanding of its functions, potentially contributing to the treatment of various diseases in the future.
